# Metagenomic Sequencing Identified Specific Bacteriophage Signature Discriminating between Healthy and Diarrheal Neonatal Piglets

**DOI:** 10.3390/nu15071616

**Published:** 2023-03-27

**Authors:** Zhenyu Wang, Jingjing Li, Lingyan Ma, Xiangdong Liu, Hong Wei, Yingping Xiao, Shiyu Tao

**Affiliations:** 1 College of Animal Sciences and Technology, Huazhong Agricultural University, Wuhan 430070, China; 2 State Key Laboratory of Animal Nutrition, College of Animal Science and Technology, China Agricultural University, No. 2 Yuanmingyuan West Road, Beijing 100193, China; 3 State Key Laboratory for Managing Biotic and Chemical Threats to the Quality and Safety of Agro-Products, Institute of Agro-Product Safety and Nutrition, Zhejiang Academy of Agricultural Sciences, Hangzhou 310021, China

**Keywords:** diarrhea, virus, metagenome, neonatal, *E. coli*

## Abstract

Neonatal diarrhea is one of the most severe diseases in human beings and pigs, leading to high mortality and growth faltering. Gut microbiome-related studies mostly focus on the relationship between bacteria and neonatal diarrhea onset, and no research study has investigated the role of the gut virome in neonatal diarrhea. Here, using metagenomic sequencing, we characterized the fecal viral community of diarrheal and healthy neonatal piglets. We found that the viral community of diarrheal piglets showed higher individual heterogeneity and elevated abundance of *Myoviridae*. By predicting the bacterial host of the identified viral genomes, phages infecting Proteobacteria, especially *E. coli*, were the dominant taxa in neonatal diarrheal piglets. Consistent with this, the antibiotic resistance gene of *E. coli* origin was also enriched in neonatal diarrheal piglets. Finally, we established a random forest model to accurately discriminate between neonatal diarrheal piglets and healthy controls and identified genus *E. coli-* and genus *listeria*-infecting bacteriophages, including psa and C5 viruses, as key biomarkers. In conclusion, we provide the first glance of viral community and function characteristics in diarrheal and healthy neonatal piglets. These findings expand our understanding of the relationship among phages, bacteria and diarrhea, and may facilitate the development of therapeutics for the prevention and treatment of neonatal diarrhea.

## 1. Introduction

Diarrhea is one of the most important biological events in human beings and piglets, particularly during infancy. Globally, neonatal diarrhea is the one of the leading causes of death in children and accounts for over half a million deaths in children under the age of 5 [1,2]. Other direct consequences of neonatal diarrhea in children include growth faltering, malnutrition and cognitive impairment [3,4]. In piglets, neonatal diarrhea leads to decreased feed intake, watery stool, and more seriously, growth cessation or death. Thus, understanding the cause of neonatal diarrhea is the first step towards diagnosis and intervention.

In recent decades, large-scale epidemiologic studies have identified lots of bacteria as the leading cause of diarrhea, mainly including *Shigella* spp., *Salmonella* spp. and *E. coli* [5,6]. These pathogens mainly inhabit the GIT and induce diarrhea by reshaping the gut environment, thus resulting in the activation of inflammation signals and alteration in host immunity. Recently, virus-like particles (VLPs), referred to as “gut dark matter”, have been gradually recognized as another dominant community inhabiting the gastrointestinal tract, and the majority are classified as phages [7]. According to life cycles, four types of phage life cycles (including lytic, temperate, pseudo-lysogenic and bacterial budding life cycles) have been reported, and lytic and temperate phages are the two major forms in the GIT [8]. Phages are widely distributed in the intestinal lumen, in the mucosal layer and in the proximity of their bacterial host, indicating profound phage–bacteria interaction. In most cases, this interaction is exhibited in a form named phage–bacteria coevolution, which acts as one of most important drivers of ecological and evolutionary dynamics in bacteria–phage meta-communities [9]. The literature has shown that several types of modes of coevolution, including arms race and negative frequency-dependent selection, are mainly observed between lytic phages and bacterial hosts in a spatially structured manner, whereas positive frequency-dependent selection is ubiquitous between temperate and filamentous phages and bacteria [9,10,11]. One of the most important systems involved in defense and counter-defense between bacteria and phages is the CRISPR-Cas system. Recent studies have also revealed that there exists coevolution in the CRISPR-Cas system, as bacteria can acquire “memory” of past infections, while phages can overcome CRISPR-Cas immunity with protospacer or PAM mutation [12,13]. Moreover, horizontal gene transfer (HGT) has been shown to be another form of phage–bacteria interaction. Numerous studies have revealed that phage genomes encode various antibiotic resistance- and virulence factor-related genes and are capable of transferring this genetic material to its bacterial host, thus causing the divergence of the genetic pool of the whole community [14,15]. Therefore, phage–bacteria interaction is one of most crucial drivers influencing community diversity, structure, and phenotypic and genotypic traits. To date, emerging studies have correlated phages with the onset of diseases such as IBD or colitis [16,17,18]. However, little information is available about the relationship between virus and neonatal diarrhea, and information is still limited.

Our laboratory has previously profiled the gut virome landscape of healthy and diarrheal weaning piglets using bulk metagenomics and meta-transcriptomics [19]. To the best of our knowledge, this is the first study to investigate the relationship between diarrhea and gut virome in pigs using a culture-independent method. Moreover, we show that the virus infecting *E. coli* was enriched in diarrheal weaning piglets. However, little knowledge is available about the original source of this gut virome difference between diarrheal and healthy weaning piglets. Thus, we examined the gut virome in the feces of healthy neonatal piglets and piglets with diarrhea. To the best of our knowledge, our study is the first one to systematically analyze the landscape of intestinal viral signatures of neonatal piglets of both heathy and diarrhea statuses with function potential, phylogenetic tree establishment, virus–bacteria interaction prediction and a random forest model, which provides the first glance of the gut virome landscape in the early life of piglets, thus providing fundamental clues for future intervention to improve the gut healthy status and growth performance output.

## 2. Materials and Methods

### 2.1. Animal Experiment

The newborn piglets used in this study were obtained from Guangxi Province, China. A total of 30 litters were used in the present study. One diarrheal piglet and one healthy piglet were selected from each litter immediately after partition. These pregnant sows received no antibiotic administration and exhibited no health issues throughout the pregnancy. Sows were fed standard corn–soybean meal diet that met the requirement of NRC 2012. These newborn piglets only had access to breast milk to avoid any dietary effect. Fresh fecal samples were collected from selected newborn piglets (30 diarrheal piglets and 30 health piglets). All selected piglets and sows did not receive any antibiotics, probiotics or prebiotics prior to sample collection. Piglets with liquid and watery feces for at least two consecutive days were classified as diarrheal piglets, while piglets showing no diarrhea or other diseases were classified as healthy piglets. Collected stool samples were stored at −80 °C.

### 2.2. DNA Extraction and Metagenomic Sequencing

Total DNA was extracted from fecal samples using E.Z.N.A.^®^ Viral DNA Kit (Omega Bio-tek, Norcross, GA, USA) according to the manufacturer’s protocol. A high-quality DNA sample (OD260/280 = 1.8~2.2, OD260/230 ≥ 2.0) was used to construct the sequencing library. Metagenomic libraries were prepared using TruSeqTM Nano DNA sample preparation Kit by Illumina (San Diego, CA, USA) using 1 μg of high-quality DNA. DNA end repair, A-base addition and adaptor ligation were performed according to Illumina’s protocol. Libraries were size-selected for target DNA fragments of ~400 bp on 2% Low Range Ultra Agarose, followed by PCR amplified using Phusion DNA polymerase (NEB) for 15 PCR cycles. Metagenomic sequencing was performed by Shanghai Biozeron Biotechnology Co., Ltd. (Shanghai, China). All samples were sequenced in pair-end 150 bp (PE150) mode using the Illumina platform.

### 2.3. Virus Identification

After removing adaptors, raw reads were subjected to the removal of low-quality reads using Trimmomatic (version 0.36) with parameters “ILLUMINACLIP:adapters.fa:2:30:10 SLIDINGWINDOW:4:15 MINLEN:75” [20]. Then, reads were mapped to the pig genome (Sus scrofa 11.1) using bwa (version 0.7.12-r1039) to remove host contamination [21]. Each sample was assembled using megahit (version 1.1.1-2-g02102e1) using default parameters [22]. Following assembly, contigs ≥ 10 kb were piped through VirFinder (version 1.1), VirSorter2 (2.0) and IMG (blastn mode) to identify viral contigs [23,24,25]. These employ different strategies to identify viral contigs; therefore, they were used to supplement each other to identify more viral contigs from the assembly. In brief, VirFinder relies on sequence signatures (k-tuple word frequencies) that distinguish viral from host sequences to predict viral contigs. VirSorter2 uses a three-step strategy to perform viral contig identification. VirSorter2 first identifies and extracts features from input sequences, and each sequence is then independently scored using a set of classifiers customized for individual viral groups. Finally, these cores are aggregated into a single prediction score as the output. Contigs with VirFinder score > 0.7 and *p* value ≤ 0.05, or identify ≥ 90% (VirSorter2) and coverage ≥ 75% (IMG) were considered putative viral contigs for downstream analysis. Given there may have existed identical or highly similar viral contigs among the identified viral sequences catalog, all viral sequences were de-replicated into viral operational taxonomic units (vOTUs) with pairwise alignment using mummer (version 4.0.0) with 95% identity and 70% coverage [26]. The longest sequence of each cluster was selected as the representative sequence, and a total of 12,458 dereplicated viral contigs were finally retained.

### 2.4. Viral Taxonomy Classification

For each viral contig, ORFs were predicted using prodigal (--meta mode; version 2.6.3) [27]. The resulting nucleotide sequences were translated into protein sequences and used as input for VPF-class to perform hmmsearch against the given hmms (VPFs) file to obtain a taxonomy classification [28]. In brief, VPF-class classifies viral contigs into different taxonomy levels based on orthologous viral proteins identified from a set of previously classified viral protein families in the IMG/VR database. A random forest classification model was constructed using the “randomforest” package with default parameters. Model performance metrics including accuracy, F1-score and AUC-ROC were calculated. The phylogenetic tree was recovered using VIPtree (version 1.1.2) [29].

### 2.5. Functional Annotation of Viral Contigs

Predicted gene catalogs were annotated using VIBRANT to annotate auxiliary metabolic genes (AMGs) [30]. VIBRANT uses KEGG annotations to classify potential AMGs. Specifically, KEGG annotations under the “metabolic pathway” category, as well as “sulfur relay system”, were considered. Manual inspection was used to remove non-AMG annotations. All AMGs were associated with a KEGG metabolic pathway map. Moreover, the gene catalog was annotated against the eggNOG database using eggNOG-mapper (v2.0) [31,32]. eggNOG-mapper relies on the eggNOG database of ortholog groups (OGs), covering thousands of bacterial, archaeal and eukaryotic organisms. It employes precomputed phylogenies inferred for each OG to efficiently refine orthology and minimize the transferring of annotations from putative in-paralogs. Carbohydrate active enzyme and antibiotic resistance were also profiled against the dbCAN2 and CARD databases using DIAMOND [33,34,35].

### 2.6. Host Prediction

Host prediction was performed using two bioinformatic methods as follows: (1) host CRISPR spacers; (2) genome similarity. First, microbial genome files of the Refseq database were downloaded from NCBI. CRISPR spacers in microbial genomes were detected using minCED (https://github.com/ctSkennerton/minced (accessed on 9 September 2019). Phage genomes were aligned to the detected CRISPR spacer catalog using BLASTN with cut-offs (alignment length ≥ 30 bp, ≥97% nucleotide identity, ≥97% query coverage and e value ≤ 10^5^) [36]. Additionally, a direct comparison between phage genomes and host genomes was performed using BLASTN to identify shared nucleotide sequences (alignment length ≥ 30 bp, ≥97% nucleotide identity, ≥97% query coverage and ≤10^5^).

### 2.7. Statistics Analysis

The R package vegan (2.6.2) was used to calculate alpha diversity (richness and Shannon index) and beta diversity (Bray–Curtis dissimilarity). Community heterogeneity was calculated using the microbiome package (1.18.0). The Wilcoxon rank test was used to analyze alpha diversity and community heterogeneity. Permutational multivariate analysis of variance (PERMANOVA) was employed to calculate statistical significance for principal coordinate analysis (PCOA) based on Bray–Curtis metrics. Differential viral family and vOTU and metabolic pathways were identified using the edgeR package. *p* < 0.05 was considered statistically significant.

## 3. Results

### 3.1. Viral Contig Statistics

To figure out the initial landscape of the gut virome in neonatal piglets, 30 healthy and 30 diarrheal piglets were selected following partition. Various virus-like particle (VLP) enrichment methods are available, and differences have been observed among these extraction and enrichment methodologies. Thus, we used bulk metagenomic sequencing to avoid any bias. The average sequencing depth was 68,951,770 reads per sample after adaptor removal and quality control. The de novo assembly-based approach was used to recover the viral contigs of piglets. A total of 12,297 vOTUs (non-redundant virus sequences) were retained after initial assembly, 10 k length filtering and vOTU dereplication. The final average length was 26,870 bp, and the longest was 461,967 bp. Of these vOTUs, after we employed blastn to align this viral contig catalog to the IMG/VR database, only 1581 vOTUs (12.86%) had a hit with previously reported viruses with 90% identity and 75% coverage thresholds (Table S1). Then, we used checkV to evaluate the completeness and contamination of these viral contigs. A total of 436 complete and 448 high-quality viral genomes were identified, accounting for around 7% of the total viral contig catalog (Figure S1). Meanwhile, 106 viral contigs of 436 complete viral genomes were identified as potential prophages based on checkV, while no prophage was identified in non-complete genomes.

### 3.2. Taxonomy Composition of Piglet Gut Virome

**Figure 1:**
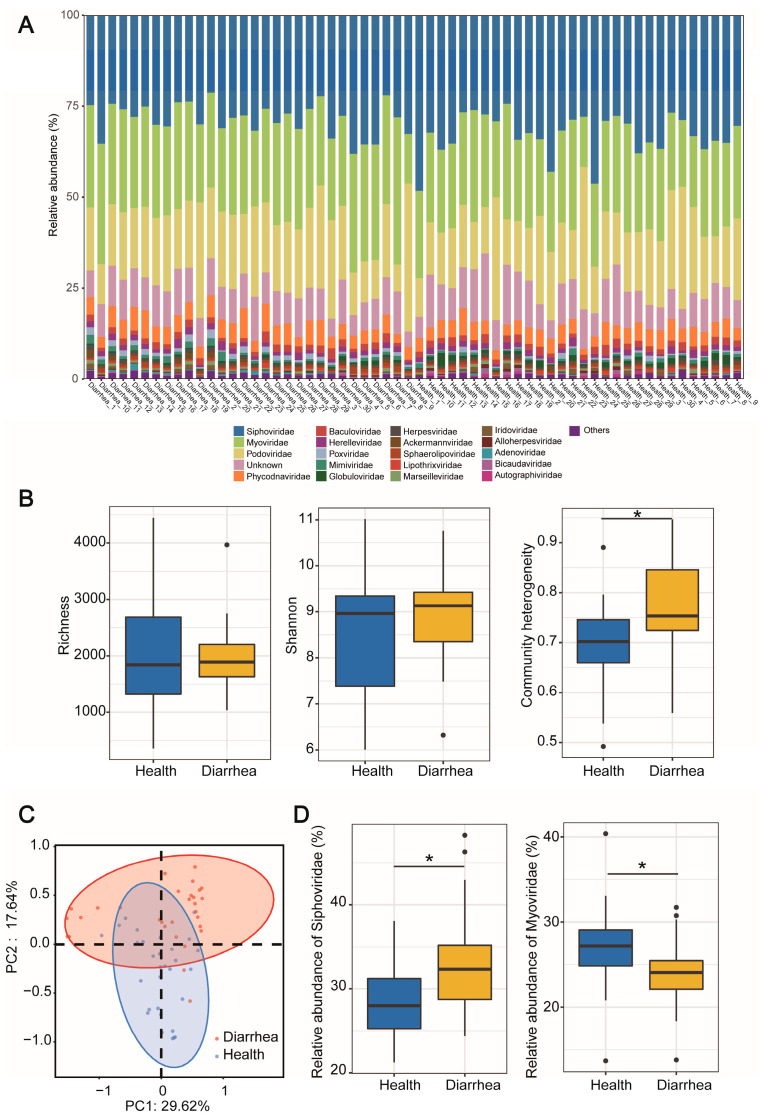
Composition, diversity and structure of gut virome in healthy and diarrheal piglets. (**A**) The relative abundance of the top 20 viral families. (**B**) Alpha diversity and community heterogeneity in healthy and diarrheal neonatal piglets. (**C**) PCOA metrics of viral community based on Bray–Curtis distance. (**D**) Relative abundance of differential viral families in healthy and diarrheal neonatal piglets. * *p* < 0.05. n = 30.

To figure out the taxonomy composition of neonatal piglets, we subjected our constructed vOTU catalog to taxonomy classification using VPF-class. The majority of taxonomically classified vOTUs were assigned to families of bacteriophages (dsDNA and ssDNA prokaryotic viruses). At the family level, the classified vOTUs were mainly assigned to *Siphoviridae*, *Myoviridae*, *Podoviridae*, *Herelleviridae* and *Globuloviridae*, accounting for, on average, 75% of the total viral community. Eukaryotic viruses were mainly composed of *Phycodnaviridae*, *Baculoviridae*, *Poxviridae* and *Mimiviridae*. Of course, around 10% of vOTUs remained unassigned (Figure 1A). The vOTU distribution showed that vOTU4465 and vOTU5808 were found in all samples by setting the count cut-off to 100 based on TPM abundance. Meanwhile, 1236 vOTUs were shared by 90% of the piglets. On the other hand, 11,061 vOTUs were shared by over 90% individuals with abundance less than 1.

### 3.3. Viral Diversity and Structure Differences between Gut Virome of Diarrheal and Healthy Piglets

We further aimed to investigate the diversity and structure of fecal viral communities. The communities exhibited no differences between healthy and diarrheal piglets in alpha diversity (Shannon index and observed species) at both the vOTU level and the family level (Figure 1B and Figure S2A). However, we observed higher community heterogeneity in the diarrheal group at the vOTU level but not at the family level (Figure 1B). Then, we compared the community structure based on PCOA metrics and found that the viral community showed a clear difference between healthy and diarrheal piglets along the PC1 and PC2 axes (*p* < 0.05, PERMANOVA; Figure 1C). The litter effect showed minimal impact on the overall viral community structure (*p* = 0.221, PERMANOVA; Figure S2C). Then, we employed edgeR to identify differential viruses between healthy and diarrheal piglets. At the family level, *Siphoviridae* and *Globuloviridae* were higher in healthy piglets, while higher abundance of *Myoviridae* and *Poxviridae* was observed in diarrheal piglets (Figure 1D and Figure S2B).

### 3.4. Virus–Host Interaction Differs between Diarrheal and Healthy Piglets

**Figure 2:**
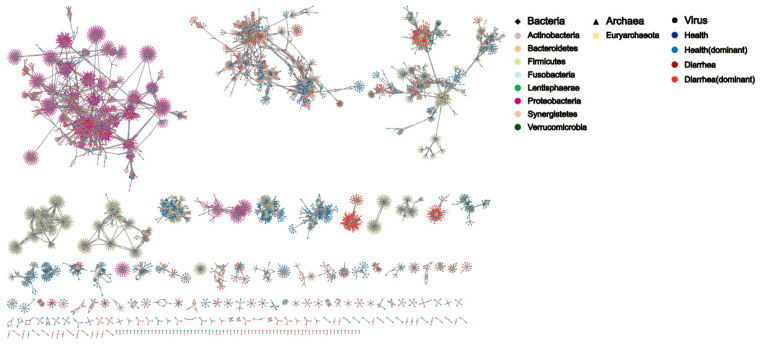
Bacteria–virus interaction network. The shape of the node represents different host taxonomy. The color within each kingdom category represents different taxonomy. Lines mean that a specific host was infected by a virus.

Predicting the bacterial host is important for understanding host–virus interaction. As the majority of our identified viral contigs were prokaryotic viruses, especially bacteriophages, we further predicted virus-infected hosts based on CRISPR spacers and genome nucleotide identity. Of note, 9820 out of 12,297 vOTUs (79.8%) were found not to infect any bacterial host. Of the remaining 2477 vOTUs, around two-thirds of vOTUs (1629/2477) were predicted to infect a single bacterial host. A small portion (14/2477) of vOTUs were predicted to infect over 10 different bacterial species (Table S2). On the other hand, members of Proteobacteria, Bacteroidetes and Firmicutes were the top three phyla predicted to be the hosts of viruses, accounting for over 90% of the total hosts (Figure S3A). At the genus level, *E. coli* accounted for over 30% of predicted bacterial hosts, followed by *Bacteroides* and *Parabacteroides* (Figure S3B). Following this, we established the virus–host network to reflect the virus–host interaction pattern. In diarrheal piglets, we observed that Proteobacteria were identified as the hosts of the majority of vOTUs. However, the majority of vOTUs identified in healthy piglets infected Bacteroidetes and Firmicutes (Figure 2).

### 3.5. Functional Annotation and Phylogenetic Tree of Differential Viral Contigs

**Figure 3:**
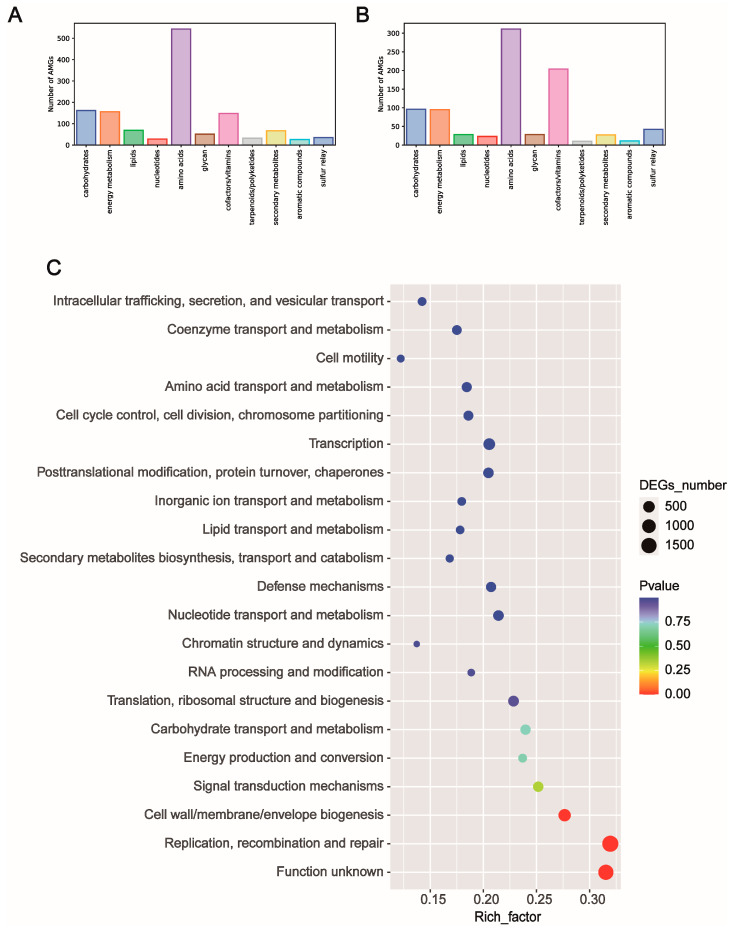
Functional potential of identified viral genome in healthy and diarrheal neonatal piglets. (**A**) Auxiliary metabolic gene (AMG) distribution in healthy neonatal piglets. (**B**) Auxiliary metabolic gene (AMG) distribution in diarrheal neonatal piglets. (**C**) COG pathway enrichment analysis using differential COG genes identified between healthy and diarrheal neonatal piglets.

Numerous viruses encode AMGs to affect various host metabolic processes. Given that distinct community structures were observed between healthy and diarrheal piglets, we assessed the AMG profiles and differences between healthy and diarrheal piglets. Overall, 11 putative AMG pathways (carbohydrate, energy metabolism, lipids, nucleotides, amino acids, glycan, cofactors/vitamins, terpenoids/polyketides, secondary metabolites, aromatic compounds and sulfur relay) were identified based on VIBRANT. Genes involved in amino acid metabolism were mainly enriched in healthy piglets (Figure 3A). Of the enriched amino acid metabolic pathways, cysteine and methionine metabolism was the main metabolic pathway. Viral AMGs related to cofactors/vitamins were higher in diarrheal piglets than in healthy piglets (Figure 3B). By mapping predicted gene catalogs to clusters of orthologous groups (COGs) embedded in the eggNOG database, the most enriched differential pathway was functional unknown, followed by cell wall/membrane/envelope biogenesis and replication, recombination and repair, signal transduction mechanisms, and energy production and conversion (Figure 3C). With regard to pathways enriched in diarrheal piglets, carbohydrate transport and metabolism, nucleotide transport and metabolism, cell wall/membrane/envelope biogenesis and replication, nucleotide transport and metabolism, and recombination and repair were the main identified pathways (Figure S4). Concerning antibiotic resistance, the AMR genes of *Staphylococcus aureus*, *Mycobacterium tuberculosis*, *E. coli*, *Neisseria gonorrhoeae* and *Paenibacillus* spp. were the top five most abundant. Of note, the gene related to E. coli in diarrheal piglets was two times higher than that of healthy piglets (Figure S5A). In addition, the total CAZy number was higher in diarrheal piglets than in healthy piglets. Specifically, a higher number of enzymes belonging to the GH and GT families were observed in diarrheal piglets (Figure S5B).

**Figure 4:**
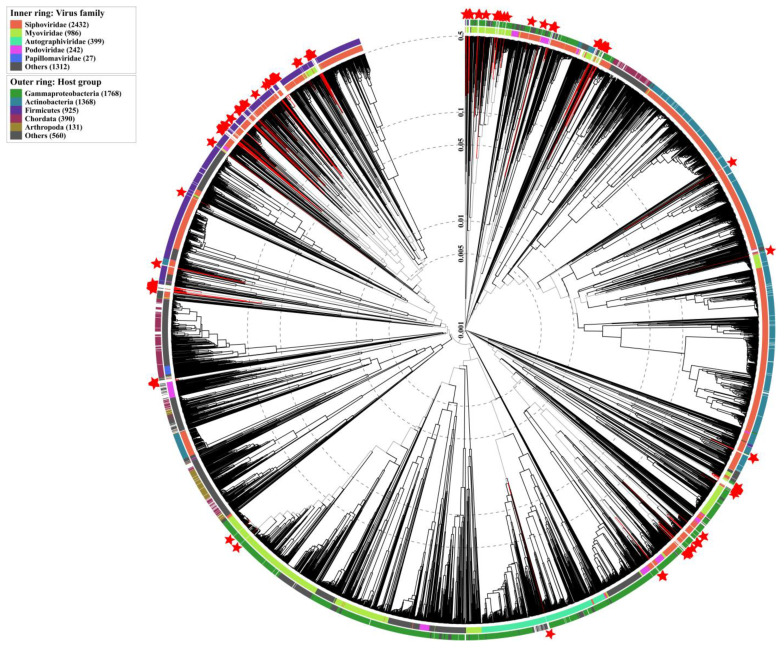
Phylogenetic tree of identified viral genomes. The inner ring represents the taxonomy classification of each clade. The outer ring represents the predicted host of each clade. A clade labeled with a pentagram means that this virus is aligned with a known virus in the IMG/VR database.

The viral genome is highly variable across different families, compared with nucleotide sequences, and it is more accurate to construct the phylogenetic tree using the amino acid sequences of the identified viral genome. To avoid the effect of genome fragmentation and contamination resulting from the assembly, the phylogenetic tree of the identified viral contigs was constructed using VIPtree. We found that the majority of the identified viral contigs with reported taxonomy classification in the IMG/VR database were classified as *Siphoviridae* and *Myoviridae*. Regarding the hosts of these identified viral contigs, *Myoviridae* mainly infected Proteobacteria, but *Siphoviridae* also infected Actinobacteria and Firmicutes other than Proteobacteria (Figure 4).

### 3.6. Viral Biomarkers Discriminating between Diarrheal Piglets and Healthy Piglets

**Figure 5:**
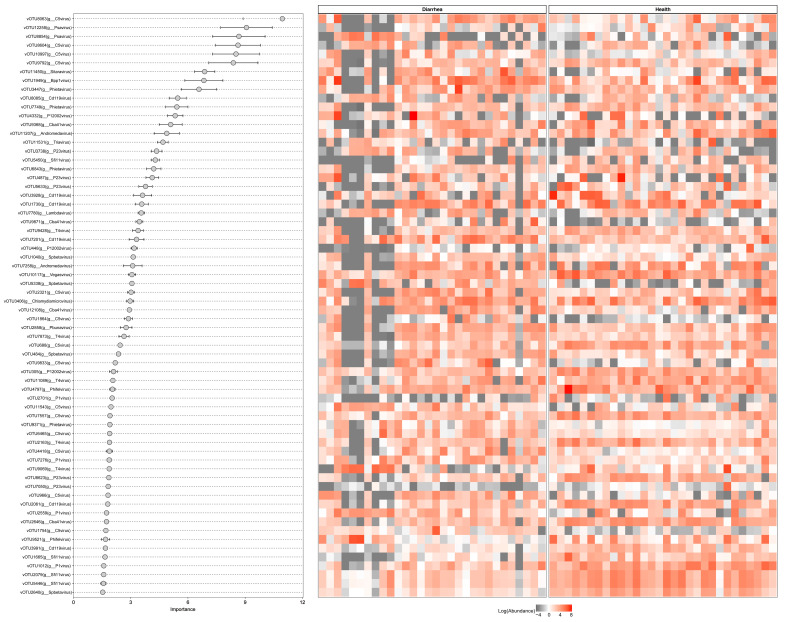
Random forest classification model identifying specific signature discriminating between healthy and diarrheal neonatal piglets. The left panel shows the top 30 viruses ordered by importance of contribution to the model. The right heatmap shows the scaled abundance distribution in each sample.

We next sought to establish a random forest classification model to identify the viral biomarkers discriminating between healthy and diarrheal piglets. To avoid the negative effect of genome fragmentation and artificial assembly, we only retained the complete viral genome identified with CheckV as input for model establishment. The Randomforest function embedded in the randomforest package was used to establish a classification model with default parameters. The established classifier exhibited 88.33% accuracy and 88.52% F1-score in terms of distinguishing healthy from diarrheal piglets (Figure S6). On the other hand, the ROC-AUC was 0.635. Then, we selected the top 30 viral contigs within the established model according to their importance. vOTU102, classified as a genus C5 virus, was the most important viral contig. Moreover, we identified several vOTUs belonging to genus psa and genus C5 viruses among the top 10 vOTUs (Figure 5).

## 4. Discussion

Neonatal diarrhea is the one of commonest causes of death in mammals. However, the majority of neonatal diarrhea-related studies focus on the bacterial community in the gastrointestinal tract. Despite the high abundance of the gut viral community, little is known about the relationship between the gut virome and neonatal diarrhea. Thus, we hypothesized that the gut viral community may be different in healthy and diarrheal neonatal piglets. In the present study, we profiled the gut viral community using metagenomic sequencing, and we found that neonatal diarrheal piglets showed higher individual heterogeneity than the healthy controls and a distinct community structure. In addition, elevated abundance of *Myoviridae* and *Poxviridae* was enriched in diarrheal piglets. Consistent with this, bacteriophages infecting *E. coli* were favored in diarrheal piglets. The random forest classification model also identified several *listeria-* and *E. coli*-infecting viruses as important biomarkers discriminating between healthy and diarrheal neonatal piglets.

Specifically, we first characterized the viral community in healthy and diarrheal neonatal piglets. Using bulk metagenomic sequencing, we reconstructed 12,297 viral genomes longer than 10 kb, 12.86% (1581/12,297) of which had homologs in the IMG/VR database. Moreover, over 90% of the identified viral genomes were predicted to be incomplete, suggesting that the majority of the gut viral community remains to be identified. Thus, we employed VPF-class to classify the identified viral contigs at the family rank based on amino acid similarity. In agreement with the results of the human gut virome, we identified several dominant gut viral families (dsRNA viruses and bacteriophages) across all samples, including *Siphoviridae*, *Myoviridae* and *Podoviridae* [37,38]. By comparing the gut virome of different individuals, we further figured out that the gut virome of piglets was highly individual specific. Only around 10% (1236/12,297) of the identified vOTUs were shared by over 90% of all samples, but this portion represented an asymmetric, larger proportion (average 32.20%) of the relative abundance of the individual gut virome. This is inconsistent with a previous study in a healthy human cohort, in which a small subset of identified viral contigs was proposed as a persistent personal virome (PPV) that occupied a large proportion of sequencing reads per sample [37,38,39]. Overall, we confirmed that the gut virome of neonatal piglets was highly individual specific.

We hypothesized that diarrhea or other disturbances may alter viral communities in neonatal piglets. In line with this, for the first time, significantly high heterogeneity of the viral community was observed in diarrheal piglets compared with the healthy controls. According to the ecological theory, disturbances generally lead to the stochastic assembly of ecological communities [40,41]. Moreover, this phenomenon is commonly observed in human IBD cohorts and murine colitis models. Furthermore, consistent, elevated abundance of Caudovirales phages, especially *Myoviridae*, was observed in both IBD patients and diarrheal neonatal piglets [16,17,18]. Therefore, disturbed viral communities characterized by high heterogeneity and elevated abundance of specific viral families may participate in the pathogenesis of diseases.

Generally, the bacteriophages in the intestine are integrated into the bacterial genome as lysogenic prophages [42,43]. Thus, addressing bacteria–phage interactions is essential to understand the effect of disturbed viral communities on neonatal diarrhea. Here, we figured out that the phages in diarrheal piglets favored the Proteobacteria phylum as bacterial hosts, especially *E. coli*. Moreover, we previously observed this in diarrheal weaned piglets. Similarly, *Escherichia phage* and *Enterobacteria phage* were shown to be enriched in UC patients, emphasizing the potential role of *E. coli* genome-integrated phages in diarrhea [16]. This phenomenon could have several underlying explanations. Generally, lytic phages attack their bacterial host, which results in the lysis of the bacterial cell. However, temperate and filamentous phages go through a totally different life cycle, in which they instead modulate host metabolism, pathogenesis or environmental fitness by manipulating relevant gene expression or introducing gene fragments with HGT [8]. In the present study, neonatal piglets showed individual specific gut viral communities representing different susceptibility to stress or selection pressure [44,45,46,47]. Under stress, *E. coli* genome-integrated temperate phages may be induced by environmental signals in the GIT. Phage excision and outbreak could act as regulators of bacterial pathogenesis, thus activating inflammation signals or reshaping the gut environment, which could facilitate the outgrowth of *E. coli* and induce the onset of diarrhea. However, we could not verify this hypothesis due to the limitation of the bioinformatic tools available to predict phage–bacteria interaction in specific species or lineages. Of note, we identified that several phages infecting *E. coli* and *listeria* were among the top 10 most important viral biomarkers by establishing a classification model and further validated the important roles of *E. coli* and *E. coli*-infecting phages in the onset of diarrhea. Bacteria have been widely used as biomarkers to predict the prognosis of diseases or discriminate between disease subjects and healthy controls in early phases, and our findings further emphasize the importance of pathogen-infected phages in diarrhea and reveal that specific phages could serve as informative markers for the early diagnosis of diarrhea.

In addition, to the best of our knowledge, we provide the first glimpse of AMG distribution and differences between healthy and diarrheal neonatal piglets. Viral AMGs related to carbon metabolism, replication, recombination and repair, nucleotide transport and metabolism, and cell wall/membrane/envelope biogenesis were the main identified categories. Viruses have been shown to engage in carbon metabolism in environments such as marine and soil environments [48,49,50]. Upon host starvation and nutrient limitation, which are very common for piglets going through diarrhea, viruses could reprogram host carbon metabolism by increasing metabolic fluxes and finally increasing energy production [51,52]. Specifically, research has demonstrated that *E. coli* could promote the metabolic flux from pyruvate to acetyl-CoA, thus toward energy production with the TCA cycle [53]. Consequently, this metabolism reprogramming of *E. coli* may result in sufficient energy supply for genome replication, including both *E. coli* and genome-integrated phages, and thus towards the rapid production and excision of phages. On the other hand, pig milk and diet often contain high contents of starch, lactose, and oligo- and polysaccharides. The enrichment of GH and GT in diarrheal piglets may contribute to the degradation of these nutrients, which could release ATP for replication or cell well synthesis.

## 5. Conclusions

Overall, to the best of our knowledge, we provide the first report of gut virome differences between healthy and diarrheal neonatal piglets. We found that the viral community showed high individual heterogeneity and distinct community structures compared with the healthy controls. Surprisingly, we observed that phages predicted to infect pathobionts (mainly *E. coli*) were heightened in diarrheal neonatal piglets. Since phages infect bacteria at strain-specific resolution, this elevated abundance of phages infecting *E. coli* may serve as an important proxy for the strain-level resolution of disease-causing bacteria. Additionally, we successfully established a random forest model to classify diarrheal and healthy neonatal piglets with high accuracy and identified *E. coli*- and *listeria*-infecting phages as potential markers, which could be useful for developing a diagnosis of neonatal diarrhea in early stages. However, our research does have limitations: We provide no mechanistic information about how elevated *E. coli*-infecting phages induce the onset of diarrhea. Moreover, we did not figure out how *E. coli*-infecting phages interact with their host, *E. coli*, and impact the bacterial community and host healthy status. Nonetheless, we think our research output could contribute to the final elucidation of the onset of neonatal diarrhea by introducing the phage as one of the potential factors.

## Data Availability

The datasets supporting the conclusions of this article are available at the NCBI Sequence Read Archive (SRA) repository under accession number PRJNA847006.

## Supplementary Materials

The following supporting information can be downloaded at: https://www.mdpi.com/article/10.3390/nu15071616/s1, Figure S1: Genome completeness of identified viral genomes; Figure S2: Diversity and relative abundance of differential viral family in healthy and diarrheal piglets. (A) alpha diversity of gut virome at family level. (B) The relative abundance of differential viral families. (C) PCoA metrics of gut virome in healthy and diarrheal neonatal piglets with color indicates same litter; Figure S3: Bacteria host distribution of identified viral genomes. (A) The predicted bacteria phylum distribution of identified viral genomes. (B) The predicted bacteria host of identified viral genomes at genus level; Figure S4: Upregulated COG pathway in diarrheal piglets. Color indicates the adjusted *p* value. The size of circular shows the number of enriched genes in corresponding pathway; Figure S5: Antibiotics resistance gene and CAZy enzymes profile in healthy and diarrheal piglets. (A) The host distribution of annotated antibiotics resistance genes in healthy and diarrhea piglets. (B) The CAZy enzymes profile in healthy and diarrheal neonatal piglets; Figure S6: Confusion matrix and ROC curve of established random forest model. (A) Confusion matrix of established random forest model. (B) ROC curve of established random forest model; Table S1: novelty of identified viral genomes; Table S2: Predicted host genome and species number of identified viral genomes; Table S3: Taxonomy classification of predicted bacteria host of viral genomes.
