# Supplementary material for: Metagenomic Sequencing Identified Specific Bacteriophage Signature Discriminating between Healthy and Diarrheal Neonatal Piglets
Source: Nutrients. 2023 Mar 27;15(7):1616. doi: 10.3390/nu15071616 (PMC10097093; doi:10.3390/nu15071616)
Supplement: Supplementary file 1 [file nutrients-15-01616-s001.zip › Supplementary file/Supplementary figures.pdf]

Metagenomic Sequencing Identified Specific Bacteriophage Signature Discriminating between Healthy and Diarrheal Neonatal Piglets

Zhenyu Wang [1,2], Jingjing Li [1], Lingyan Ma [3], Xiangdong Liu [1], Hong Wei [1], Yingping Xiao [3,*] and Shiyu Tao [1,*]

1 College of Animal Sciences and Technology, Huazhong Agricultural University, Wuhan 430070, China

2 State Key Laboratory of Animal Nutrition, College of Animal Science and Technology, China Agricultural University, No. 2 Yuanmingyuan West Road, Beijing 100193, China

3 State Key Laboratory for Managing Biotic and Chemical Threats to the Quality and Safety of Agro-Products, Institute of Agro-Product Safety and Nutrition, Zhejiang Academy of Agricultural Sciences, Hangzhou 310021, China

Figure S1: Genome completeness of identified viral genomes.

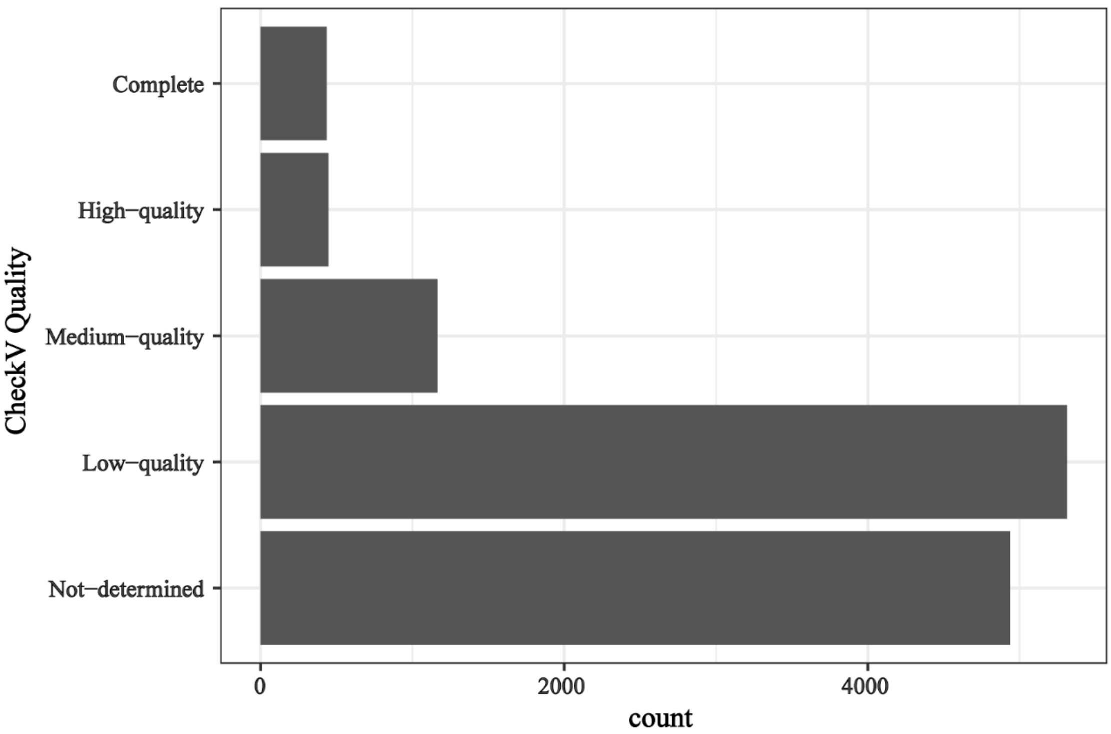

Figure S2: Diversity and relative abundance of differential viral family in healthy and diarrheal piglets. A. alpha diversity of gut virome at family level. B. The relative abundance of differential viral families. C. PCoA metrics of gut virome in healthy and diarrheal neonatal piglets with color indicates same litter.

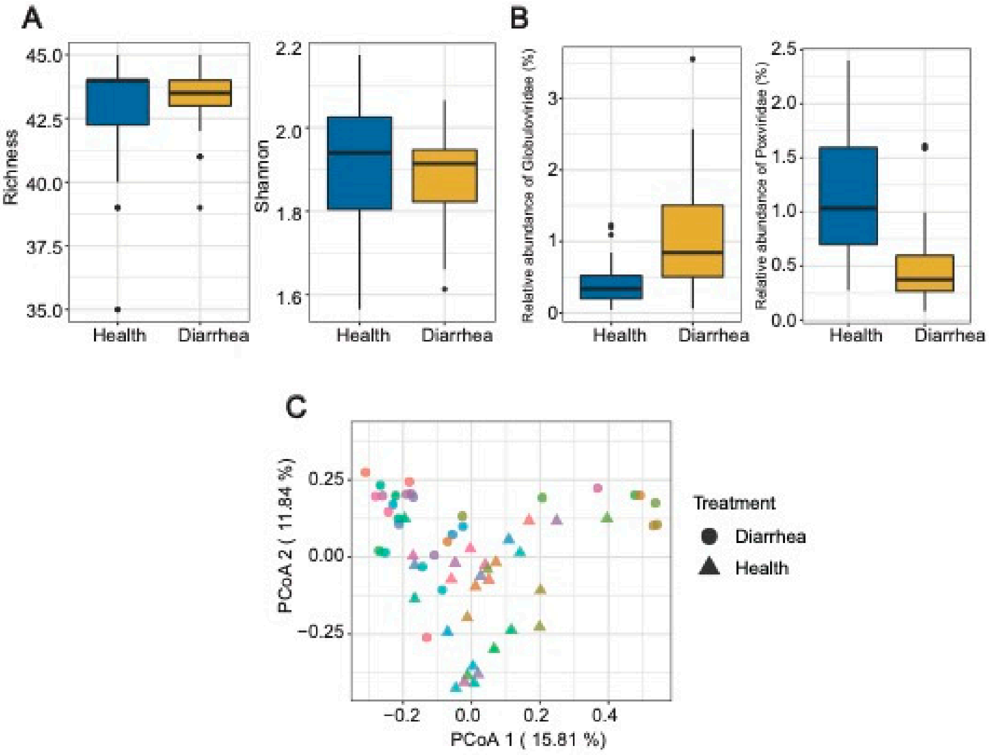

Figure S3: Bacteria host distribution of identified viral genomes. A. The predicted bacteria phylum distribution of identified viral genomes. B. The predicted bacteria host of identified viral genomes at genus level.

A

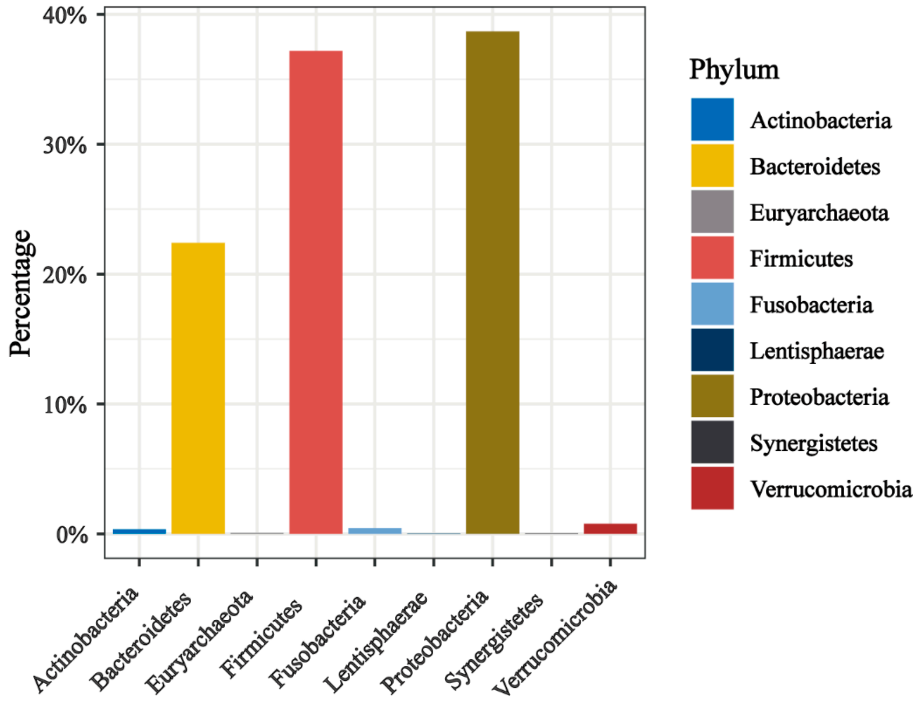

B

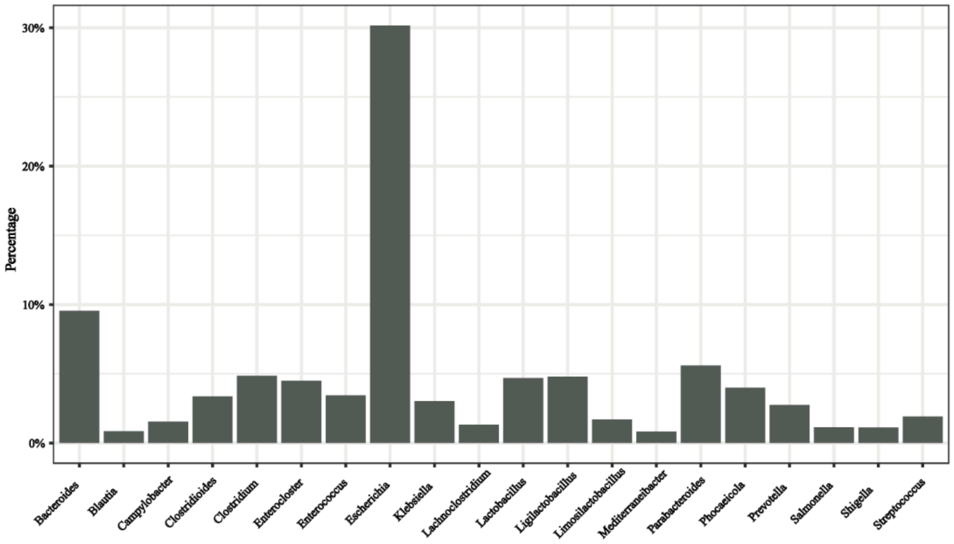

Figure S4: Upregulated COG pathway in diarrheal piglets. Color indicates the adjusted p value. The size of circular shows the number of enriched genes in corresponding pathway.

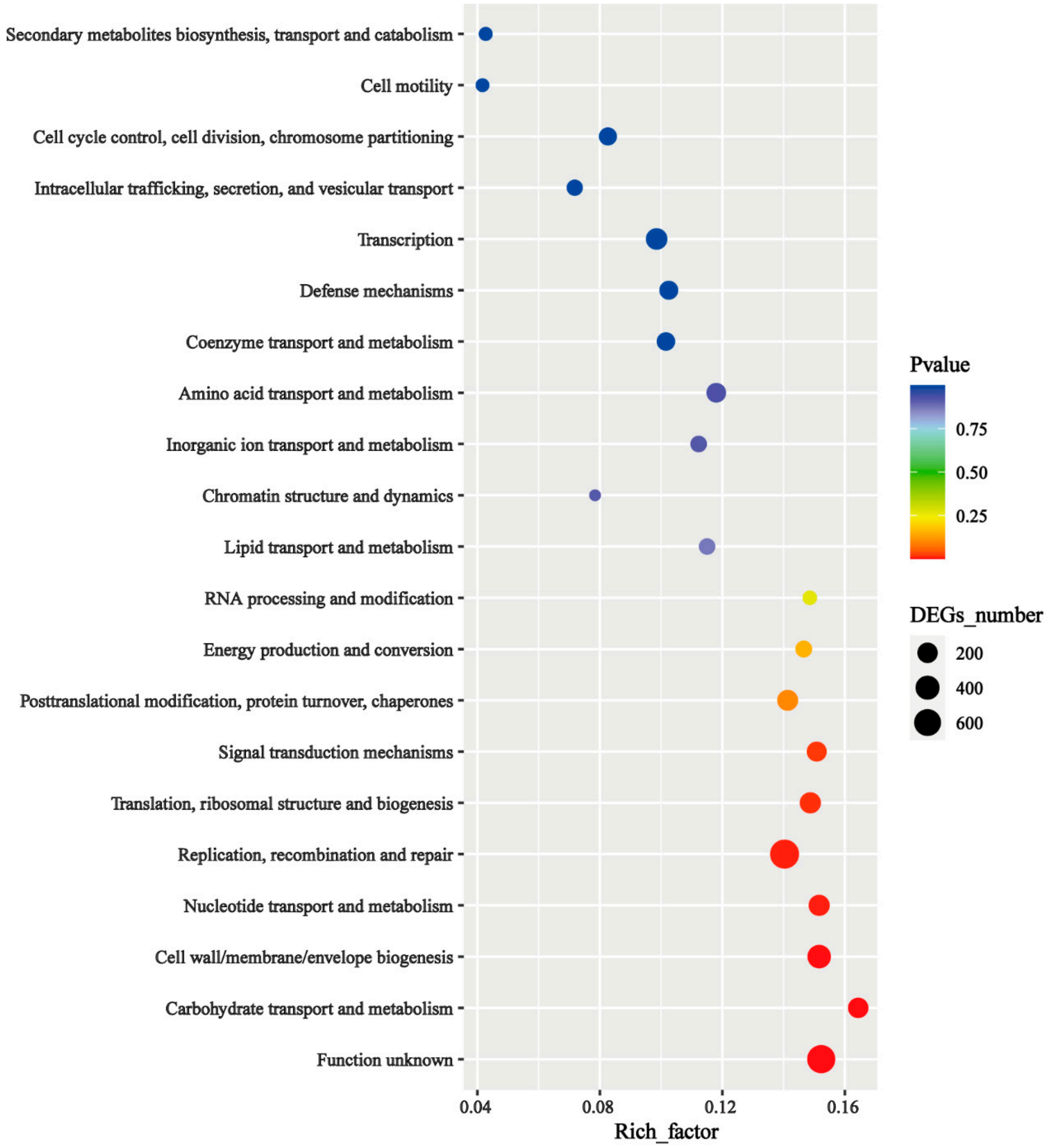

Figure S5: Antibiotics resistance gene and CAZy enzymes profile in healthy and diarrheal piglets. A. The host distribution of annotated antibiotics resistance genes in healthy and diarrhea piglets. B. The CAZy enzymes profile in healthy and diarrheal neonatal piglets.

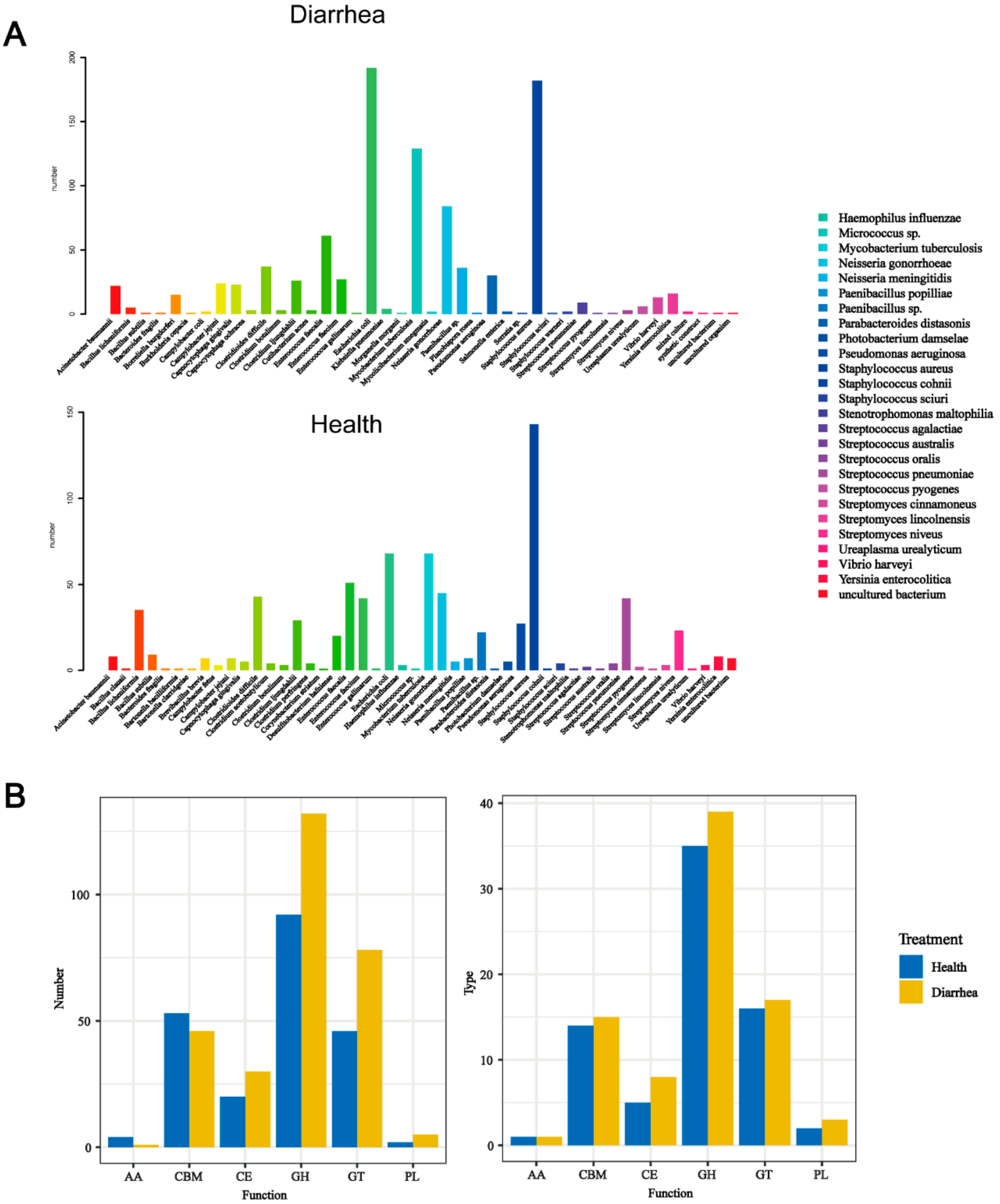

Figure S6: Confusion matrix and ROC curve of established random forest model.

A. Confusion matrix of established random forest model. B. ROC curve of established random forest model.

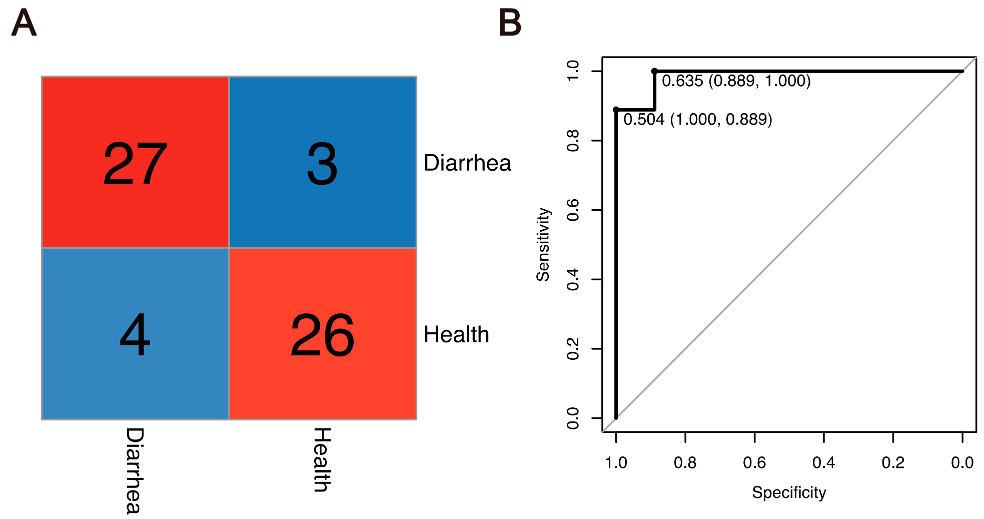

Table S1: Novelty of identified viral genomes

Table S2: Predicted host genome and species number of identified viral genomes

Table S3: Taxonomy classification of predicted bacteria host of viral genomes
